# Novel insights into the regulation of cyclooxygenase-2 expression by platelet-cancer cell cross-talk

**DOI:** 10.1042/BST20140322

**Published:** 2015-08-03

**Authors:** Melania Dovizio, Sara Alberti, Angela Sacco, Paloma Guillem-Llobat, Simone Schiavone, Thorsten J. Maier, Dieter Steinhilber, Paola Patrignani

**Affiliations:** * Department of Neuroscience, Imaging and Clinical Sciences, Section of Cardiovascular and Pharmacological Sciences, “G. d'Annunzio University”, Chieti, Italy; † Center of Excellence on Aging (CeSI), “G. d'Annunzio” University Foundation, Chieti, Italy; ‡ Institutes of Pharmaceutical Chemistry, Goethe-University, Frankfurt am Main, Germany

**Keywords:** colon cancer cells, cyclooxygenase 1 (COX-1), cyclooxygenase 2 (COX-2), epithelial mesenchymal transition, metastasis, platelets

## Abstract

Platelets are activated by the interaction with cancer cells and release enhanced levels of lipid mediators [such as thromboxane (TX)A_2_ and prostaglandin (PG)E_2_, generated from arachidonic acid (AA) by the activity of cyclooxygenase (COX)-1], granule content, including ADP and growth factors, chemokines, proteases and Wnt proteins. Moreover, activated platelets shed different vesicles, such as microparticles (MPs) and exosomes (rich in genetic material such as mRNAs and miRNAs). These platelet-derived products induce several phenotypic changes in cancer cells which confer high metastatic capacity. A central event involves an aberrant expression of COX-2 which influences cell-cycle progression and contribute to the acquisition of a cell migratory phenotype through the induction of epithelial mesenchymal transition genes and down-regulation of E-cadherin expression. The identification of novel molecular determinants involved in the cross-talk between platelets and cancer cells has led to identify novel targets for anti-cancer drug development.

## Introduction

Platelets are chief effector cells in haemostasis but they are also inflammatory cells. In fact, they play a central role in the cross-talk with stromal and immune cells leading to their activation which is crucial in the progression of malignant, inflammatory and metabolic diseases [1].

**Figure 1:**
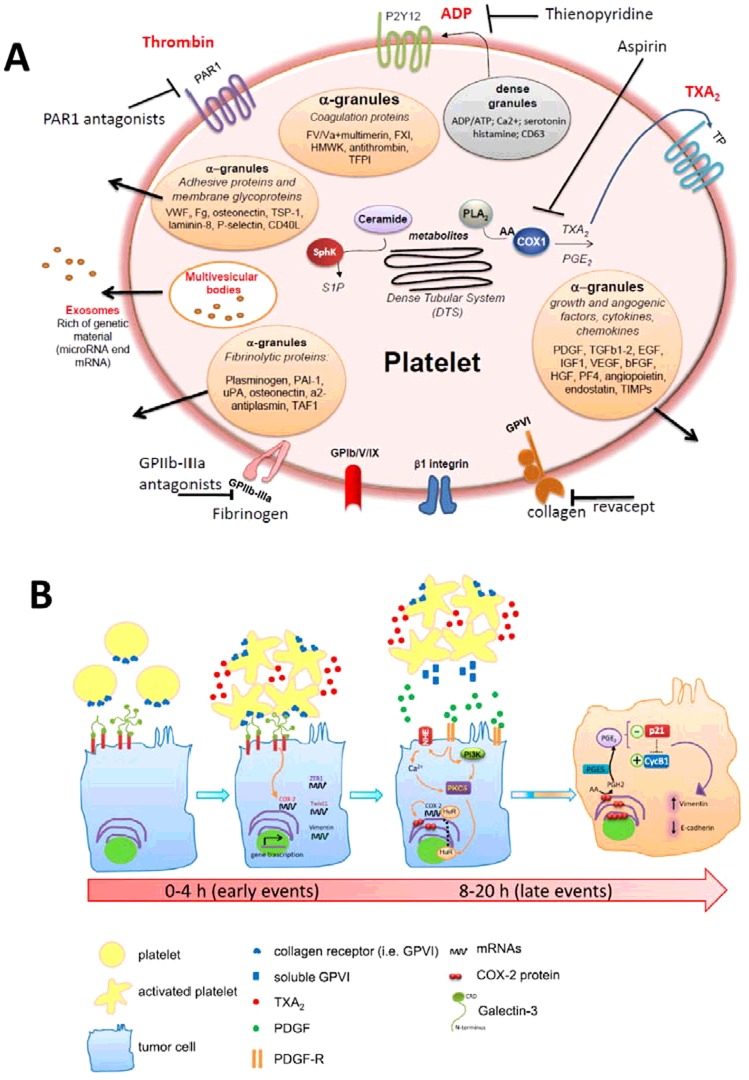
Platelets induce a pro-metastatic phenotype in colorectal cancer cells *in vitro* (**A**) Biology of platelets and pharmacological inhibitors of platelet responses. (**B**) Proposed mechanism of COX-2 overexpression in tumour cells by the interaction with platelets. Unstimulated platelets interact rapidly with tumour cells through the binding of platelet collagen receptors (in particular, GPVI) and tumour components, such as galectin-3. This early event translates into platelet activation, as demonstrated by enhanced generation of TXA_2_. Direct platelet-tumour cell interaction is associated with enhanced mRNA expression of COX-2 (but not COX-2 protein) and EMT-inducing transcription factors, such as zinc finger E-box binding homeobox 1 (ZEB1) and Twist1 and the mesenchymal marker vimentin. Later, platelet aggregates detach from tumour cells, possibly as a consequence of the shedding of platelet GPVI receptors and acquire the capacity to release their α-granule products, such as PDGF. The release of PDGF is associated with COX-2 mRNA stabilization via NHE–PI3K/PKCδ-dependent nucleo-cytoplasmic translocation of the mRNA-stabilizing protein HuR and COX-2 protein synthesis. In HT29 cells, overexpressed COX-2 and enhanced generation of PGE_2_ emanate mitogenic and survival signalling pathways through the down-regulation of p21^WAF1/CIP1^ and the up-regulation of cyclin B1 as well as of EMT-inducing transcription factors and mesenchymal markers, such as vimentin, in association with repression of epithelial markers, such as E-cadherin. Adapted from [18]: Dovizio, M., Maier, T.J., Alberti, S., Di Francesco, L., Marcantoni, E., Munch, G., John, C.M.,Suess, B., Sgambato, A., Steinhilber, D. and Patrignani, P.(2013) Pharmacological inhibition of platelet-tumour cell cross-talk prevents platelet-induced overexpression of cyclooxygenase-2 in HT29 human colon carcinoma cells. Mol. Pharmacol. **84**, 25–40.

Upon activation, platelets undergo a shape change and release lipid mediators [such as thromboxane (TX)A_2_ and prostaglandin (PG)E_2_, generated from arachidonic acid (AA) by the activity of cyclooxygenase (COX)-1] and dense and α-granule content (i.e., ADP and growth factors, chemokines and proteases respectively; Figure 1A). These effects are associated with conformational changes in their major integrin receptor glycoprotein (GP) IIb/IIIa (αIIbβ3) leading to the aggregation of platelets to other platelets via fibrinogen (Fb) thus leading to the formation of a haemostatic plug [2].

Traditional anti-platelet agents, such as aspirin and thienopyridines, affect platelet function through the irreversible inhibition of platelet COX-1 activity and blocking the ADP receptor P2Y12, respectively [3,4] (Figure 1A). Drugs targeting proteinase-activated receptor (PAR)1 are in clinical development as anti-platelet agents [5,6]. PAR1 is a G-protein coupled receptor (GPCR) which is activated by thrombin-dependent proteolytic cleavage of the receptor N-terminus to reveal a cryptic tethered ligand motif [7]. Once cleaved, the receptor is believed to undergo intramolecular rearrangement and the tethered ligand initiates signalling. Recently, Food and Drug Administration (FDA) has approved vorapaxar as first-in-class PAR-1 antagonist for the reduction in thrombotic cardiovascular events in patients with a history of heart attack or with peripheral arterial disease [www.fda.gov/NewsEvents/Newsroom/PressAnnouncements/ucm396585]. Other novel anti-platelet agents in clinical development include revacept, a dimeric Fc fusion protein with the IgG part and the extracellular domain of the human glycoprotein VI (GPVI) platelet receptor, which interferes in the collagen-induced activation of platelets [8]. Revacept prevents the first steps of collagen-mediated platelet adhesion and the consecutive platelet aggregation and platelet activation associated with vascular damage. Differently from other anti-platelet agents, it has been claimed that revacept prevents collagen-mediated platelet interaction with the atherosclerotic endothelium without influencing bleeding time [8].

Novel mechanisms by which platelets play a role in cardiovascular disease and tumour progression and metastasis may involve their capacity to release microparticles (MPs) and exosomes [9]. These platelet-derived MPs are approximately 0.1–1.0 μm in diameter in humans and express P-selectin (CD62P) and GP IIb-IIIa. They adhere to and activate a variety of cells, such as endothelial cells, leucocytes and other platelets [10,11]. Exosomes, range in size from 0.04 to 0.1 μm, arise from the internal membrane vesicles of multivesicular bodies and granules in platelets [11] (Figure 1A). Unlike MPs, exosomes do not share a similar surface phenotype of activated platelets. However, both MPs and exosomes are known to carry and deliver cellular signals, suggesting a potential role in platelet-derived signalling. Exosomes can be released also from cancer cells and can be detected in the systemic circulation [12]. They house a cytosol-like protein repertoire, together with unique mRNA and miRNA species [13] resembling that of the origin tumour, thus they could be used as surrogate diagnostic markers for biopsy profiling [14], extending its utility to screening asymptomatic populations. miRNAs are endogenous approximately 22 nt RNAs that can play important regulatory roles in animals and plants by targeting mRNAs for cleavage or translational repression [15]. Despite the growing evidence on the role of platelet-derived vesicles in physiology and pathology, it remains to disclose several aspects. In fact, very few information is available on the molecular mechanisms involved in platelet vesicular trafficking. No anti-platelet agent has been developed to interfere with vesicle formation and release.

## Role of platelet–cancer cell interaction in metastasis

Within the circulatory system, platelets guard tumour cells from immune elimination thus contributing to their survival [16]. Moreover, platelets promote cancer cell arrest at the endothelium thus facilitating their extravasation and spreading to other sites of the body [16].

Labelle et al. [17] have demonstrated that the interaction between platelets and tumour cells leads to epithelial-mesenchymal-like transition (EMT) which is a cellular phenotype that allows tumour cells to colonize distant organs. In mice, platelet-derived transforming growth factor (TGF)-β and direct platelet-tumour cell contacts synergistically activate the TGF-β/small mother against decapentaplegic protein (Smad) and NF-κB (nuclear factor kappa-light-chain-enhancer of activated B-cells) pathways in cancer cells, resulting in their transition to an invasive mesenchymal-like phenotype and enhanced metastasis *in vivo*. Inhibition of NF-κB signalling in cancer cells or ablation of TGF-β1 expression solely in platelets protects against lung metastasis *in vivo* [17].

Dovizio et al. [18] have recently shown that the interaction of human platelets with HT29 colon carcinoma cells is associated with enhanced and persistent expression of COX-2 (Figure 1B).

Two isoforms of COX (COX-1 and COX-2) have been cloned and characterized [19]. These enzymes catalyse the conversion of AA to prostanoids [PGE_2_, PGF_2α_, PGD_2_, prostacyclin (PGI_2_) and TXA_2_] [20]. COX-1 gene is considered a ‘housekeeping gene’ and it is highly expressed in platelets and gastric epithelial cells where it plays a role in causing platelet activation, via the generation of TXA_2_ and gastric cytoprotection, via the generation mainly of PGE_2_ respectively [21]. Differently, the gene for COX-2 is a primary response gene with many regulatory sites [22]. However, COX-2 is constitutively expressed in some cells in physiologic conditions, such as endothelial cells [23], where COX-2-dependent PGI_2_ (prostacyclin) is a vasoprotective pathway [24] and in pathological conditions, such as in cancer cells [25]. COX-2 overexpression, in cancer cells, occurs through post-transcriptional mechanisms in part due to altered expression of trans-acting factors that bind to AREs (AU-rich elements) and regulate the status of mRNA stability [25,26]. In particular, overexpression of the mRNA-stability factor human antigen R (HuR) and concomitant loss of the mRNA decay factor tristetraprolin (TTP) can synergistically promote enhanced COX-2 expression, in colon cancer [27]. COX-2 mRNA 3′- UTR contains binding site for some miRNAs [miRNA-response elements (MREs)] that when expressed might promote down-regulation of COX-2 by affecting COX-2 mRNA stability [28].

Enhanced expression of COX-2 in platelet–cancer cell co-cultures requires both a direct interaction and the release of platelet mediators [18] (Figure 1B).

The cellular determinants of the direct interaction between platelets and HT29 cells are platelet collagen receptors (in particular, GPVI) and tumour components, such as galectin-3. This interaction translates into enhanced transcription of COX-2 gene [18].

Wnt signalling cascade is activated in colorectal cancer [29] and it may trigger the transcription of several genes involved in tumorigenesis, such as COX-2 [30], through the accumulation of β-catenin into the nucleus. β-Catenin is a multifunctional protein serving as a major structural component of cell-to-cell adherens junctions [31]. In addition, it also acts as an important signalling molecule in the Wnt pathway that plays a key role in embryogenesis and tumorigenesis [31,32]. In the absence of Wnt signalling, the cytoplasmic level of β-catenin is kept low through interaction with a protein complex [containing GSK3β (glycogen synthase kinase 3β), axin and adenomatous polyposis coli (APC)] that can phosphorylate β-catenin and target it to ubiquitin-mediated proteasomal degradation. Activation of Wnt signalling leads to inactivation of GSK3β, resulting in cytoplasmic accumulation of β-catenin. The increase in β-catenin level is followed by its translocation into the nucleus, where in complex with members of the T-cell factor (Tcf)/lymphocyte enhancer-binding factor family of transcription factors it activates the expression of target genes, such as COX-2 [29,33].

**Figure 2:**
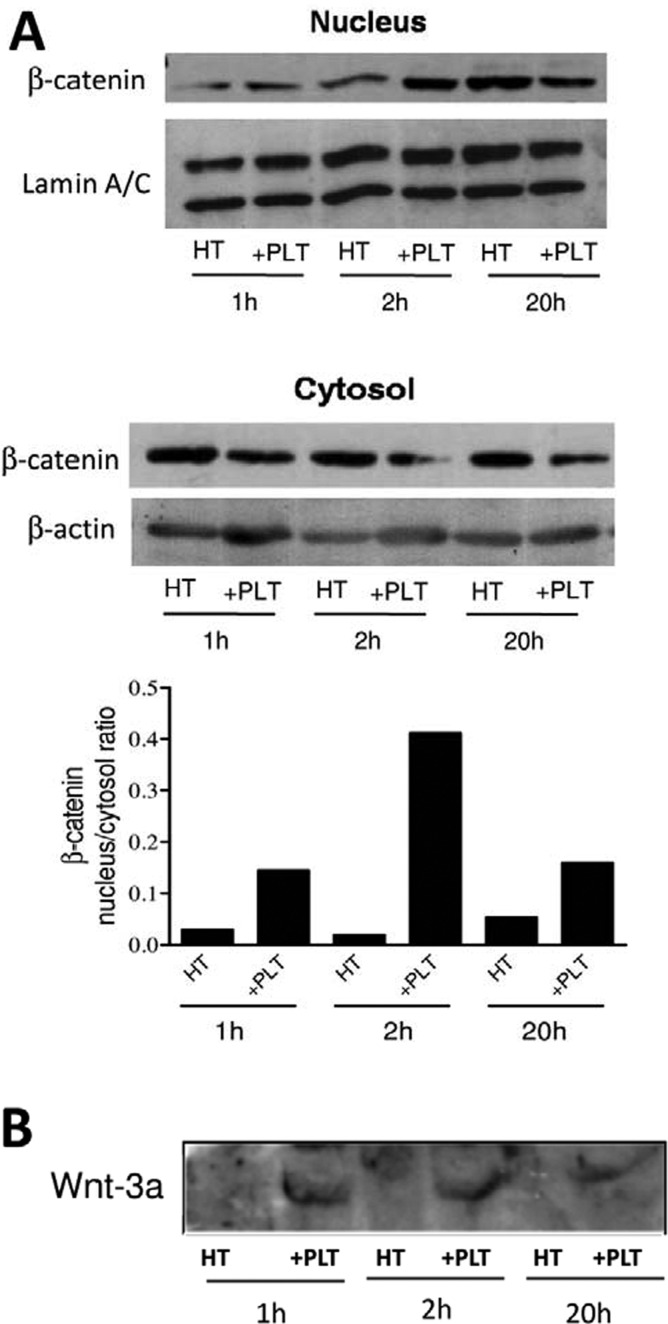
Effect of platelets on β-catenin translocation into the nucleus (**A**) β-Catenin was assessed by western blot in the cytoplasmic and nuclear fractions of HT29 cells (1×10^6^ cells) cultured alone (HT) or in the presence of platelets (+PLT), up to 20 h; quantification of attenuance (*D*) of different specific bands was calculated using laser densitometry and normalized to the *D* of β**-**actin and values were reported; lamin A and C, which are essential scaffolding components of the nuclear envelope, expression was detected in the same samples by Western blot technique.(**B**) Wnt3a levels were assessed by western blot in supernatants of HT29 cells cultured alone or with platelets.

As shown in Figure 2(A), we have found that the incubation of HT29 cells with platelets is associated with a time-dependent induction of β-catenin translocation into the nucleus (Dovizio M., Maier T.J., Steinhilber D. and Patrignani P., unpublished work).

Previous findings have shown that the canonical Wnt3a–β-catenin pathway is present in platelets and may regulate platelet function [34]. Interestingly, Steele et al. [34] found that Wnt3a is secreted from thrombin receptor agonist peptide-6 (TRAP)-activated platelets. These findings prompted us to verify whether Wnt3a was detectable in supernatant of HT29 cells cultured alone or with platelets. As shown in Figure 2(B), western blot analysis of supernatants showed that Wnt3a was not detected in HT29 cells cultured alone whereas it was detectable in cancer cells cultured with platelets (Dovizio M., Maier T.J., Steinhilber D. and Patrignani P., unpublished work).

Altogether, these results suggest that Wnt released by activated platelets leads to β-catenin translocation into the nucleus thus causing the rapid increase in COX-2 mRNA levels detected in HT29 cells cultured in the presence of platelets (Figure 1B).

Other platelet mediators, such as platelet-derived growth factor (PDGF), participate in COX-2 overexpression detected in HT29 cells cultured with platelets. It was found that PDGF leads to mRNA stabilization of COX-2. This phenomenon occurs via sodium-hydrogen exchanger (NHE)–PI3K (phosphoinositide 3-kinase)/protein kinase C (PKC)δ-dependent nucleo-cytoplasmic translocation of the mRNA-stabilizing protein HuR [18] (Figure 1B).

Taken together, these studies have identified novel molecular mechanisms triggered by platelets which result in aberrant COX-2 expression in colorectal cancer cells and open the way to innovative anti-cancer therapeutic strategies. Interestingly, it has been reported that inhibitors of galectin-3 function (β-lactose, a dominant-negative form of galectin-3, Gal-3C and anti-galectin-3 antibody M3/38) or revacept prevented platelet-dependent COX-2 overexpression in cancer cells [18]. These findings provide the rationale for testing blockers of collagen-binding sites, such as revacept and galectin-3 inhibitors in the prevention of colon cancer metastasis in animal models.

In HT29 cells, overexpressed COX-2 and enhanced generation of PGE_2_ emanate mitogenic and survival signalling pathways through the down-regulation of p21^WAF1/CIP1^ and the up-regulation of cyclin B1 [18,35] as well as of EMT-inducing transcription factors and mesenchymal markers, such as vimentin, in association with repression of epithelial markers, such as E-cadherin [18] (Figure 1B). These responses were prevented by selective inhibition of COX-2 activity by rofecoxib [18].

Similar results were obtained by affecting platelet-cancer cell interaction by revacept. However, the anti-platelet agent was more effective than rofecoxib in preventing platelet-induced mRNA changes of EMT markers [18], suggesting that direct cell–cell contact and aberrant COX-2 expression synergistically induced gene expression modifications associated with EMT (Figure 1B).

## Effect of platelet releasate on cancer cell COX-2 expression

Platelet activation leads to secretion of soluble factors, including TXA_2_ and PGE_2_, granule content and to the formation of microvesicles by shedding of membranes from the cell surface [36]. The platelet releasate comprises of a multitude of inflammatory and vasoactive substances, which can attract atherogenic leucocytes from the circulation, activate endothelial cells and stimulate vessel growth and repair by triggering vascular cell proliferation, migration and inflammation [37].

Recent progress in uncovering more than 300 proteins in the thrombin-activated platelet releasate may advance our ability to understand the events involved and responses triggered in the progression of diseases which are associated with platelet activation, such as atherothrombosis and cancer [37,38].

**Figure 3:**
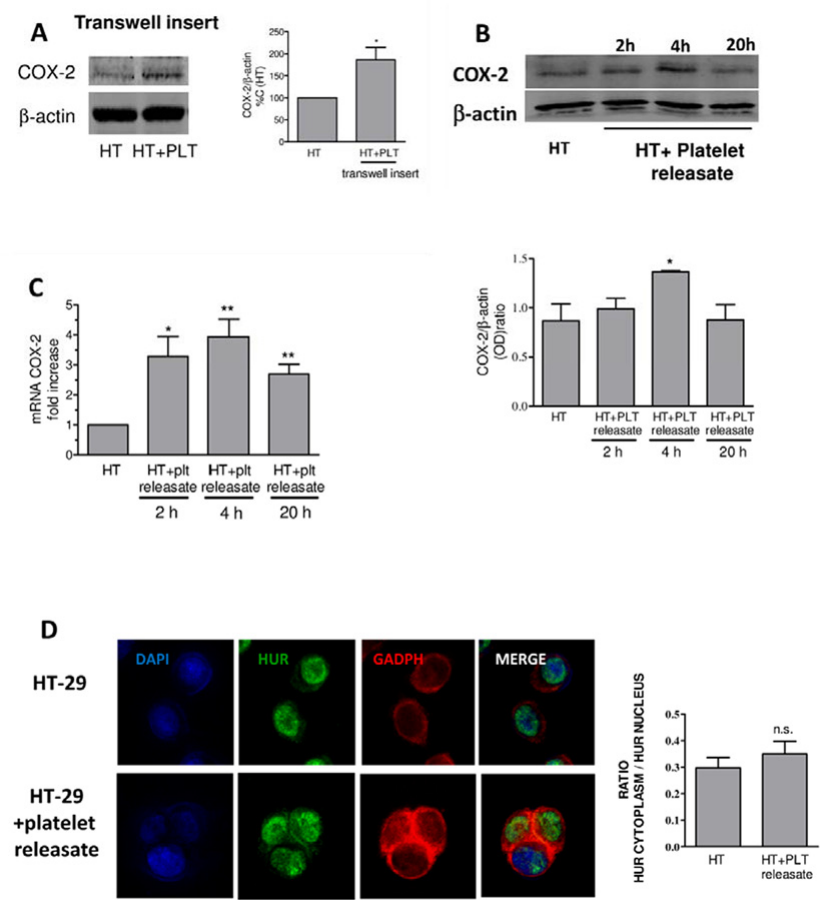
Regulation of COX-2 induction in HT29 cells by platelet releasate (**A**) HT29 cells (1×10^6^) and washed human platelets (100×10^6^) were co-cultured for 20 h using a trans-well (pore size, 0.4 μm) and COX-2 levels were assessed in HT29 cells by Western blot [18]; data are expressed as mean±S.E.M. (*n*=7), *P<0.05 compared with HT-29. (**B** and **C**) Effect of human platelet releasate on HT29 COX-2 expression; HT29 cells were cultured up to 20 h in the absence and in the presence of platelet releasate (obtained by collecting the supernatant of washed human platelets cultured for 20 h, then it was centrifuged at 750 ***g*** for 15 min) which contains soluble mediators and vesicles, i.e. MPs and exosomes; at different time-points COX-2 protein levels (**B**) and mRNA levels (**C**) were analysed by Western blot (normalized to β-actin levels) and quantitative real-time PCR (qPCR) (normalized to GAPDH) respectively, as previously described [18]; data are expressed as mean±S.E.M. (*n*=3); *P<0.05 and **P<0.01 compared with HT-29.(**D**)HuR localization was assessed by confocal microscopy analysis in HT29 cells alone (HT) or incubated with platelet releasate for 20 h; immunostaining of HuR is shown in green, GAPDH (cytoplasmic protein) in red and DAPI (nuclear marker) in blue; merge images are shown (merge); ratios between the pixel sum of HuR staining in cytoplasm and pixel sum of HuR staining in the nucleus were calculated using LAS AF software, 2.2.1, as described in [18]. Data are expressed as mean±S.E.M. (*n*=14); ns, not significant.

We studied the expression levels of COX-2 in co-cultures of HT29 cells with human platelets for 20 h performed in the presence of a transwell cell insert with a pore size of 0.4 μm (Dovizio M., Maier T.J., Steinhilber D. and Patrignani P., unpublished work). It avoids direct contact between the two cell types but permits the exchange of soluble factors, MPs and exosomes between cells. As shown in Figure 3(A), we detected enhanced protein levels of COX-2 in HT29 cells. Platelets released very low levels of TXB_2_ and PDGF, as compared with the experimental condition that allowed a direct interaction between the two cell types [18].

These results have prompted us to verify whether platelet releasate obtained by culturing washed human platelets for 20 h was able to influence COX-2 expression in HT29 cells. Thus, HT29 cells were cultured up to 20 h, in the absence and in the presence of platelet releasate. Under these experimental conditions, we found a time-dependent induction of COX-2 mRNA and protein (Figures 3B and 3C; Dovizio M., Maier T.J., Steinhilber D. and Patrignani P.). The effect was transient with a maximal up-regulation of COX-2 protein expression detected at 4 h of cell culture.

Post-transcriptional gene regulatory events, involving COX-2 mRNA decay, play a key role in the control of cellular levels of COX-2 mRNA [25]. Among them, there is HuR translocation from the cytoplasm into the nucleus and its binding within 3′-UTR of COX-2 mRNA. Immunofluorescence localization of HuR was examined by confocal microscopy analysis in HT29 cells cultivated with platelet releasate (Figure 3D) [18]. Platelet releasate did not affect HuR trafficking to the cytoplasm of cancer cells. This result may explain the transient induction of COX-2 in response to platelet releasate that we detected (Figures 3B and 3C). Differently, when platelets were allowed to interact directly with HT29 cells, nucleo-cytoplasmic translocation of HuR was detected and this was associated with a sustained induction of COX-2 protein levels [18]. The role of PKCδ in the export of HuR from the nuclear compartment was evidenced using rottlerin, which has been shown to interfere specifically with PKCδ-triggered HuR phosphorylation, in a cell-free HuR phosphorylation assay [39].

These results may implicate that the induction of COX-2 by platelet releasate did not involve post-transcriptional regulation of gene expression but rather an effect of platelet releasate on COX-2 gene transcription. Taken together, these data suggest a novel regulatory pathway involved in COX-2 induction by platelet-derived factors which does not require a direct interaction between platelets and cancer cells.

## Concluding remarks

Both epidemiological and experimental studies have reported that non-steroidal anti-inflammatory agents (NSAIDs; tNSAIDs and coxibs) reduce the risk of developing colonic tumours [40,41]. This effect is consistent with an effect of NSAIDs on cancer growth and metastasis through the inhibition of COX-2, in stromal and transformed cells [38,41]. However, the promising use of coxibs in chemoprevention was halted abruptly due to the detection of enhanced cardiovascular risk [41,42]. Novel strategies to obtain a tissue-specific inhibition of COX-2 expression or activity in tumour cells might represent an innovative direction to develop safer therapeutic tools to fight cancer development and metastasis.

A growing body of evidence supports the central role of platelets in metastasis [16–18]. Platelets are activated by the interaction with cancer cells and release several soluble mediators (such as PGE_2_, TXA_2_, PDGF, TGF-β and Wnt3a) and MPs/exosomes [18,43]. These platelet-derived products induce several phenotypic changes in cancer cells which confer high metastatic capacity. A central event involves an aberrant expression of COX-2 which influences cell-cycle progression and contributes to the acquisition of a cell migratory phenotype through the induction of EMT genes and down-regulation of E-cadherin expression [18].

The traditional anti-platelet agent, low-dose aspirin, has been proven effective in the reduction in incidence and mortality of different types of cancer by restraining the development of metastasis [44–47]. The identification of novel molecular determinants involved in the cross-talk between platelets and cancer cells has led to enlighten novel targets for anti-cancer drug development.

## Abbreviations

AA: arachidonic acid
COX: cyclooxygenase
EMT: epithelial-mesenchymal-like transition
GPVI: glycoprotein VI
GP IIb-IIIa: glycoprotein IIb-IIIa
GSK3β: glycogen synthase kinase 3β
HuR: human antigen R
NHE: sodium-hydrogen exchanger
MP: microparticle
NF-κB: nuclear factor kappa-light-chain-enhancer of activated B-cells
NSAID: non-steroidal anti-inflammatory agent
PAR: proteinase-activated receptor
PDGF: platelet-derived growth factor
PG: prostaglandin
PI3K: phosphoinositide 3-kinase
PKC: protein kinase C
TGF: transforming growth factor
TX: thromboxane
